# Effects of Valproic Acid on Cerebral Nutrient Carriers' Expression in the Rat

**DOI:** 10.3389/fphar.2018.01054

**Published:** 2018-09-21

**Authors:** Aniv Mann Brukner, Tamir Ben-Hur, Asaf Honig, Dana Ekstein, Sara Eyal

**Affiliations:** ^1^Transporter Laboratory, Institute for Drug Research, School of Pharmacy, The Hebrew University of Jerusalem, Jerusalem, Israel; ^2^Department of Neurology, The Agnes Ginges Center for Human Neurogenetics, Hadassah Medical Center, Hebrew University of Jerusalem, Jerusalem, Israel

**Keywords:** valproic acid, antiepileptic drugs, carriers, transporters, *Slc2a1*, *Glut1*, histone deacetylase

## Abstract

**Objective:** The antiepileptic drug valproate has been shown to affect the expression of carriers for essential compounds and drugs in extracerebral tissues. The aim of the current study was to evaluate *in vivo* the effect of valproate treatment on the cerebral expression of carriers and selected genes of the blood-brain barrier (BBB) in the rat.

**Methods:** Male Wistar rats were treated daily for 7 days by intraperitoneal injections of valproate (75, 150, or 300 mg/kg/day) or the vehicle. mRNA was isolated from the cerebral cortex and the hippocampus. Transcript levels of 37 genes were measured using a customized gene expression assay. Quantitative histone acetylation was evaluated by western blotting. Glucose6-phosphate (G6P) tissue levels were used as a surrogate of cerebral glucose concentrations.

**Results:** Valproate treatment was associated with significant reduction (up to 22%; *P* < 0.05) in cortical and hippocampal claudin 5-normalized *Slc2a1* (Glut1) mRNA expression. G6P levels were not significantly altered, but were correlated with *Slc2a1* transcript levels (*r* = 0.499; *P* < 0.02). None of the other 36 screened genes were significantly affected by valproate. Cortical histone hyperacetylation indicated cerebral activity of valproate on a major pathway regulating gene expression (*P* < 0.02).

**Significance:** The effect of valproate on nutrient carriers appears to be tissue-specific and even brain area-specific. If validated in humans, the changes in Glut1 expression might have clinical implications in positron emission tomography (PET) imaging. Further studies are required for elucidating the relevance of these findings to the clinic.

## Introduction

Valproic acid (VPA) is a broad-spectrum antiepileptic drug, whose exact mechanisms of therapeutic and adverse effects are still not fully understood (Tomson et al., 2016b).

We and others have shown in extracerebral tissues that VPA can regulate the expression of membrane carriers involved in the cellular uptake and efflux of hormones, nutrients, and medications. For example, VPA affected the expression of P-glycoprotein (*ABCB1*) in tumor cell lines and in rat livers (Eyal et al., 2006; Hauswald et al., 2009) and that of folate receptor α (FRα; *FOLR1*) in pregnant mice (Meir et al., 2016) and in a human placental cell line (Rubinchik-Stern et al., 2015). Furthermore, VPA modulated the expression of genes encoding carriers for nutrients, including folate and glucose, in term human placentas (Rubinchik-Stern et al., 2018).

Here, we examined whether the effects of VPA on carrier expression are replicated in the brain. We conducted an initial screening which included key cerebral nutrient carriers and components of the blood-brain barrier (BBB), given its central role in controlling the levels of essential compounds and medications within the brain parenchyma.

## Materials and methods

### Animals

This study was carried out in accordance with the principles of the Basel Declaration and recommendations of National Institutes of Health guide for the care and use of Laboratory animals (publication No. 8023, revised 1978). The protocol (MD-16-14989-3) was approved by the Institutional Animal Care and Use Committee of the Hebrew University.

Rats (male Wistar, 250–300 gr, Harlan Laboratories, Rehovot, Israel) had free access to standard food and water and were maintained on a 12/12 h light/dark cycle. Handling was performed at least 3 days prior to the initiation of injections. During the study, all animals were weighed daily and checked for signs of distress. Tissue collection was performed as previously described (Mann et al., 2018).

### VPA treatment

Rats were randomly divided into four experimental groups and were treated over seven consecutive days twice daily, 8 h apart, with intraperitoneal (i.p.) injections of 2 μL/gr body weight of sodium valproate (Sigma Aldrich, St. Louis, MO, USA) in saline or the vehicle. The VPA daily doses were 75 mg/kg [equivalent of therapeutic dose in humans (Löscher, 2002)], 150 and 300 mg/kg (for dose-response relationships). The latter was previously shown to be non-toxic (Tremolizzo et al., 2002). Of note, oral administration route, which is commonly used in human patients, was not used as we wished to avoid dependency upon the animals' drinking and eating habits. In addition, i.p. administration allows us to repeat the same method as used in previously relevant publications (Eyal et al., 2006).

### Analysis of mRNA expression

RNA extraction was performed using an RNA extraction kit (Qiagen, Hilden, Germany). Gene expression analysis was measured using digital molecular barcoding technology (nCounter; NanoString, Seattle, WA, USA) (Geiss et al., 2008). Experiments were performed according to the manufacturer's instructions (Rubinchik-Stern et al., 2018). Gene targets (Supporting Table 1) were chosen based on their importance in essential compounds' transport or BBB integrity and the absence of published data on the effect of VPA on their cerebral expression *in vivo*. Raw gene expression count data were normalized against the negative and positive technical controls and the geometrical mean of three housekeeping genes: β-actin, glyceraldehyde 3-phosphate dehydrogenase (GAPDH) and hypoxanthine phosphoribosyltransferase 1 (Hprt1), whose expression was previously shown to be unaffected by VPA treatment (Zhou et al., 2016). *Slc1b2* [in humans: SLC1B1; OATP1B1 (Roth et al., 2012)] was used as negative control, since it is not expressed in the brain (Obaidat et al., 2012).

Normalized count of 50 was considered as the limit of detection. Regions and targets with similar profiles were identified using a hierarchically clustered heatmap of normalized digital counts.

### Plasma glucose concentrations

Plasma samples and representative control and treatment cortex samples were assayed using a photometric assay (detection at 540 nm) and the GLUC3 system (Cobas, Roche, Hague Road, IN, USA; catalog number 04404483 190).

### Cerebral glucose 6-phosphate concentrations

Cortical tissues were homogenized as described previously (Mann et al., 2018), with the exception of the use of phosphate-buffered saline (PBS; Biological Industries, CT, USA) solely as the medium. The colorimetric glucose 6-phosphate (G6P) Assay Kit (Abcam, Cambridge, UK; catalog number: ab83426) was used to evaluate the effect of VPA on intracellular glucose level in the cortical tissue. Samples were analyzed in duplicate. Data are reported as total G6P amount in the homogenate (nmol).

### Histone acetylation analysis

Histone extraction from cortex tissue samples was performed using histone extraction kit (Abcam, Cambridge, UK). Sodium dodecyl sulfate-polyacryl amide gel electrophoresis, followed by transfer to nitrocellulose membranes, were performed as described previously (Rubinchik-Stern et al., 2015; Mann et al., 2018). We used primary antibodies against acetylated H3 or H4 (1:500 and 1:20,000, respectively, Abcam, Cambridge, UK) and horseradish peroxidase–conjugated secondary antibodies (1:10,000, Jackson ImmunoResearch laboratories Inc., Baltimore Pike, PA, USA). β-actin (1:5,000, Abcam, Cambridge, UK) was used as internal control. Bands quantification was performed using ImageJ.

### Statistical analysis

The required number of animals was estimated based on the observed variability and effect size obtained in our recent study on the effect of VPA on carrier expression in perfused human placentas (Rubinchik-Stern et al., 2018). The Kruskal-Wallis test with Dunn's *post-hoc* test was used for comparing across groups the expression of individual genes, histone acetylation and G6P levels. Spearman's analysis was used for correlations (InStat; GraphPad, La Jolla, CA, USA). A *P* ≤ 0.05 was considered significant.

## Results

### The effect of VPA on cerebral mRNA levels

**Figure 1:**
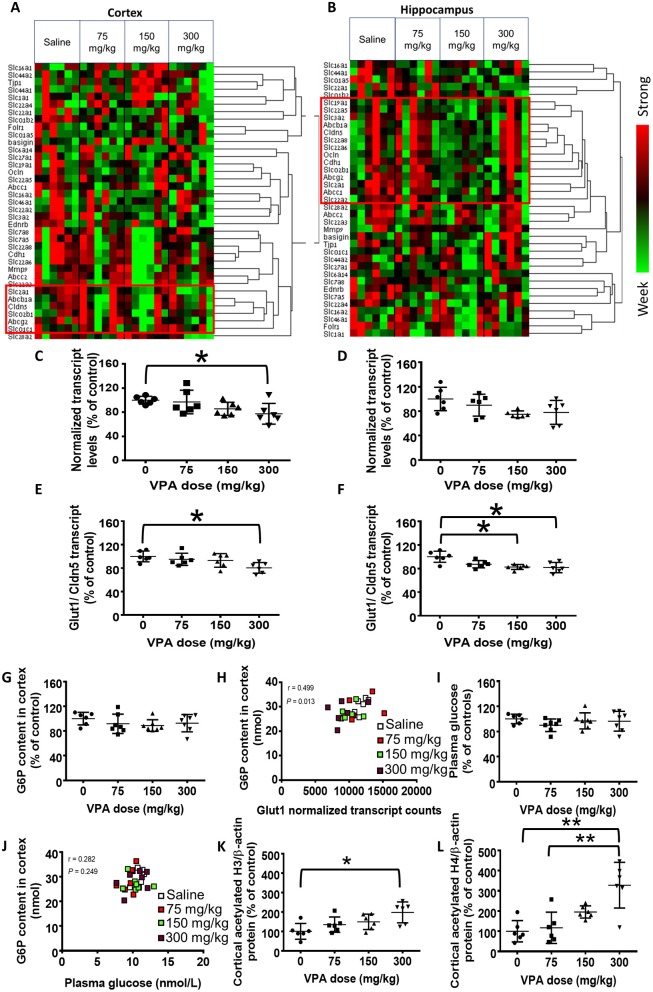
Effect of VPA on cerebral gene expression. **(A,B)** Hierarchically clustered normalized nCounter heatmaps of carriers and BBB components mRNA levels at the **(A)** cortex and **(B)** hippocampus. For each gene, expression was normalized to the geometrical mean of three housekeeping genes and the negative and positive technical controls. Each vertical column represents one animal belonging to the treatment group indicated in the upper panel. Each horizontal lane represents the normalized mRNA counts for one gene. The colors represent the expression of each gene among the different treated animals (red and green represent strong and weak expression, respectively). Red frames mark the genes which were clustered with *Glut1*. **(C,D)** nCounter gene expression analysis of cortical **(C)** and hippocampal **(D)** *Glut1* mRNA levels. **(E,F)** Cortical **(E)** and hippocampal **(F)** *Glut1* mRNA normalized to the BBB marker Cldn5. **(G)** Cortical G6P levels (nmol). **(H)** Correlation between cortical G6P levels and *Glut1* transcript counts. **(I)** Plasma glucose concentration (mM). **(J)** Correlation between cortical G6P and plasma glucose. **(K,L)** Histone acetylation in VPA-treated rats. **(K)** Cortical histone H3 acetylation. **(L)** Cortical histone H4 acetylation. Values in **C–G,I,K**, and **L** are means with SD and presented as percent of control; *P < 0.05, **P < 0.01, *N* = 6–7/group.

Cortical (Figure 1A) and hippocampal (Figure 1B) heatmaps generated from the nCounter gene expression analysis revealed an intriguing pattern of gene expression and co-regulation by VPA in the different brain structures (*n* = 6/group). For example, *Slc2a1* (Glut1) was clustered together with *Cldn5* (claudin 5) and *Abcg2* (the breast cancer protein) in both cerebral structures, but it was clustered closely only with *Abcc1* (multidrug resistance protein 1) in the hippocampus.

The expression of all housekeeping genes was not significantly altered by VPA (Table 1). As expected*, Slco1b2* expression values were below the limit of detection. The magnitude of change in the expression of most genes did not exceed 15%. Changes of >25% were observed only in the expression of nine genes: *Cdh1, Ednrb, Slc16a2, Mmp9, Slc22a4, Slc22a6, Slc22a8, Slc44a1*, and Slc7a8.

**Table 1:** Percent change in cortical and hippocampal gene expression, derived from the gene panel analysis (nCounter).

**Gene symbol (protein)**	**Cortex**				**Hippocampus**			
	**75 mg/kg**	**150 mg/kg**	**300 mg/kg**	***P***	**75 mg/kg**	**150 mg/kg**	**300 mg/kg**	***P***
*Abcb1a (MDR1a)*	100	91	98	0.885	102	91	106	0.882
*Abcc1* (Mrp1)	92	99	89	0.082	98	90	89	0.07
*Abcc2* (Mrp2)	95	85	94	0.843	79	79	84	0.594
*Abcg2* (Bcrp)	101	87	82	0.186	103	87	87	0.226
*Cdh1* (Cadherin 1)	77	N/A	55	0.402	101	N/A	155	0.523
*Cldn5* (Claudin 5)	103	92	95	0.812	102	89	95	0.786
*Ednrb* (Ednrb)	97	115	76	0.227	88	76	73	0.103
*Folr1* (FRα)	N/A	54	22	0.326	N/A	N/A	N/A	N/A
*Mmp9* (Mmp9)	127	81	78	0.829	106	118	147	0.619
*Ocln* (Occludin)	84	97	82	0.809	108	102	120	0.452
*Slc16a1* (Mct1)	104	108	96	0.125	87	98	92	0.121
*Slc16a2* (Mct8)	89	69	96	0.806	145	125	120	0.293
*Slc19a1* (Rfc)	82	88	79	0.741	117	95	120	0.261
*Slc1a1* (Eaat3)	103	106	97	0.91	95	102	100	0.817
*Slc46a1* (Pcft)	107	96	99	0.786	111	99	100	0.764
*Slc3a2* (Lat subunit)	97	88	87	0.13	95	83	86	0.051
*Slc7a5* (Lat1)	97	87	97	0.254	91	93	95	0.189
*Slc7a8* (Lat2)	89	72	98	0.217	103	97	97	0.936
*Slc6a14* (ATB^0, +^)	N/A	N/A	N/A	N/A	N/A	N/A	N/A	N/A
*Slc28a2* (Cnt2)	88	101	76	0.164	100	79	97	0.876
*Slc22a4* (Octn1)	99	109	94	0.947	75	64	91	0.054
*Slc22a5* (Octn2)	92	96	87	0.771	116	92	113	0.326
*Slc22a6* (Oat1)	55	31	57	0.579	389	69	332	0.512
*Slc22a8* (Oat3)	70	56	61	0.613	192	80	204	0.545
*Slc22a1* (Oct1)	N/A	N/A	N/A	N/A	N/A	N/A	N/A	N/A
*Slc22a2* (Oct2)	N/A	N/A	N/A	N/A	N/A	N/A	N/A	N/A
*Slc22a3* (Oct3)	N/A	N/A	N/A	N/A	N/A	N/A	N/A	N/A
*Slc44a1* (Ctl1)	104	129	83	0.079	77	69	78	0.283
*Slc44a2* (Ctl2)	100	108	97	0.197	103	97	107	0.528
*Slc27a1* (Fatp1)	100	97	104	0.926	97	96	108	0.405
*Slc2a1* (Glut1)	97	85	77	0.0414	89	74	78	0.107
*Slco1b2* (Oatp1b2)	N/A	N/A	N/A	N/A	N/A	N/A	N/A	N/A
*Slco1c1* (Oatp1c1)	99	99	99	0.98	103	103	105	0.924
*Slco2b1* (Oatp2b1)	107	96	100	0.55	104	96	99	0.809
*Tjp1* (ZO-1)	104	110	93	0.071	92	82	101	0.186
*Basigin* (Basigin)	100	108	102	0.502	101	97	110	0.418
*Slco1a5* (Oatp1a3)	N/A	N/A	N/A	N/A	N/A	N/A	N/A	N/A

*For each gene, expression was normalized to the geometrical mean of three housekeeping genes and the negative and positive technical controls. Kruskal-Wallis test, followed by Dunn's post-test. N/A – not available due to values below limit of detection; Abc, ATP-binding cassette; Slc, solute carrier; Oat, organic anion transporter; Oatp, organic anion transporter polypeptide; Oct, organic cation transporter; P-gp, P-glycoprotein; Bcrp, breast cancer resistance protein; Mrp, multidrug resistance protein; Lat, L- type amino acid transporter; Rfc, reduced folate carrier; Glut 1, glucose transporter 1; FRα (Folr1), folate receptor α; Pcft, proton coupled folate transporter; Mct, monocarboxylate transporter; Octn, organic cation/carnitine transporter; ATB^0, +^, amino acid transporter B^0, +^; Ctl, choline transporter like-protein; Fatp, fatty acid transporter protein; Eaat, excitatory amino acid transporter; Cnt, concentrative nucleoside transporter; Tjp1, tight junction protein 1; ZO-1, zonula occludens 1; GAPDH, glyceraldehyde 3-phosphate dehydrogenase*.

In this screening, the only VPA-induced alteration which was statistically significant (*P* < 0.05, *n* = 6/group) was the reduction in *Slc2a1* levels in the cortex at the highest VPA dose, compared with the saline controls (22%, *P* < 0.05; Figure 1C). The reduction in hippocampal *Slc2a1* expression was not statistically significant (*P* > 0.05, *n* = 6/group; Figure 1D).

*Glut1* is almost exclusively expressed in endothelial cells of the BBB, and each sample could have contained a different portion of endothelium. Hence, we normalize ed *Glut1*'s nCounter gene expression values to those of *Cldn5*, which is a pivotal component of the BBB, and whose levels were unaffected by VPA treatment (Table 1). This ratio was significantly lower (19%; *P* < 0.05, *n* = 6/group) in the cortices of the 300 mg/kg treatment group (Figure 1E), compared with the saline controls. Additionally, a statistically significant (*P* < 0.05, *n* = 6/group) reduction of 17% in the 150 mg/kg and 18% in the 300 mg/kg group, compared with the saline controls, was apparent in the hippocampus (Figure 1F). *Cldn5-*normalization of the levels of other carriers and BBB components did not affect the results (data not shown).

### The effect of VPA on cerebral glucose and G6P content

Since cerebral glucose concentrations were below the assay's limit of detection [used routinely for plasma glucose levels, but brain:plasma concentration ratio is ~0.2 (Mergenthaler et al., 2013),] we measured the phosphorylated, intracellular form of glucose, G6P, as a surrogate for cerebral glucose concentrations. The cortical content of G6P (nmol/mg cortex) was not significantly altered by VPA (*P* > 0.05, *n* = 6–7/group; Figure 1G). However, G6P content did positively correlate with *Glut1* mRNA levels (*P* < 0.05, *n* = 6–7/group; Figure 1H).

In order to rule out a causal effect of systemic glucose homeostasis on cerebral G6P content, we measured plasma glucose concentrations. These concentrations did not significantly differ among the treatment groups (*P* > 0.05, *n* = 6–7/group; Figure 1I) and did not correlate with the cortical G6P levels (*P* > 0.05, *n* = 6–7/group; Figure 1J).

### The effect of VPA on cerebral histone acetylation

We assessed cortical histone acetylation as a surrogate of VPA concentrations that can affect cerebral gene expression. Acetylation of both histone H3 and H4 was significantly enhanced by VPA in a dose-dependent manner, by up to 4-fold, (Figures 1K,L).

### Toxicity

Visual inspection of the rats before and after each VPA treatment revealed no unusual behavior and no signs of distress. VPA did not cause significant alteration in rats' weight, compared with the saline controls (Supporting Figure 1).

## Discussion

### VPA effects on cerebral glucose kinetics

Using gene array analysis, we demonstrated that VPA selectively downregulated cerebral *Glut1* mRNA expression, but did not significantly alter other 36 genes encoding nutrient transporters and BBB components. These effects were brain region-specific. In support of our findings, others reported a negative effect of VPA on GLUT1 expression *in vitro* (Wong et al., 2005; Wardell et al., 2009), along with direct inhibition of GLUT1-mediated cellular glucose intake (Wardell et al., 2009). Additionally, we recently demonstrated reduced GLUT1 mRNA levels in human placentas perfused *ex vivo* for 3 h with VPA (Rubinchik-Stern et al., 2018).

The lack of change in the expression of other genes examined is somewhat contradictory to previous findings in extracerebral tissues and in cell lines (Wong et al., 2005; Wardell et al., 2009). Particularly, using the nCounter analysis, we found that VPA affects the expression of multiple carriers in the human placenta (Rubinchik-Stern et al., 2018). This may point to tissue- or species-specific effect of VPA on carrier expression. Alternatively, VPA could have differentially affected various cell types or structures of the brain that express the same carrier, with tendencies that counteracted.

Cerebral G6P correlated with Glut1 transcript levels but was not significantly affected by VPA. Interestingly, VPA treatment reduced the cerebral radioactivity of ^18^F-2-deoxyglucos (^18^F-FDG) in patients with epilepsy (Leiderman et al., 1991) and in healthy volunteers (Gaillard et al., 1996). This finding was attributed to altered cerebral glucose metabolism. Our findings, especially if further validated by more sensitive and direct measurement of cerebral glucose, suggest an alternative explanation: VPA downregulates the major facilitator of glucose uptake at the BBB, Glut1 (Han et al., 2017), with potential subsequent reduction in cerebral glucose uptake. Positron emission tomography (PET) may be more sensitive than the biochemical methods to the effects of VPA on cerebral GLUT1, because in contrast to G6P, FDG-6-phosphate is not further metabolized by the glycolytic pathway and becomes trapped in the cell (Jadvar et al., 2009).

Of note, in this study, we demonstrated the effects of VPA in isolation from the disease (e.g., epilepsy), as part of our greater objective to study the effects of the disease and its medications, each in isolation from the other on carrier expression. However, we cannot rule out that studying the effect of the medication in an epilepsy animal model might have produced different results. Furthermore, we cannot rule out that different AEDs may produce different effects on carrier expression, which may also vary according to the tissue in question. These and additional concerns will be the focus of future research.

### VPA treatment increases histone acetylation

The significant elevation in the acetylation of two major histones suggests that the cerebral levels of VPA, an established inhibitor of histone deacetylases (Tomson et al., 2016a), were high enough to activate major transcriptional regulators. Epigenetic regulation of *SLC2A1* expression, mediated by VPA-induced HDAC inhibition, can result in an increase (Tanegashima et al., 2017) as well as decrease (Wardell et al., 2009) in its mRNA levels. Future studies will address the causative relationships between histone hyperacetylation and altered GLUT1 expression following exposure to VPA.

### Study limitations

We used a limited number of animals, which may have contributed to the high intersubject variability and reduced the power to detect changes in the expression of some of the genes. However, in most cases the magnitude of change was modest. Furthermore, the BBB expression of the human homologs of organic anion and organic cation transporters, which were more extensively affected by VPA, is low (Morrissey et al., 2012; Pardridge, 2015). Hence, even if the differences in gene expression in this study were considered statistically significant, their relevance, if translated to humans, is not clear. The significant effect of VPA on histone acetylation rules out poor VPA penetration into the brain and suggests that the number of animals was sufficient to detect robust effects. In addition, the use of whole-tissue homogenates could have masked some VPA effects on individual tissue types.

## Conclusions

This study demonstrates that VPA significantly reduces Glut1 expression in the brain, similarly to its effects in other tissues, but does not affect other carriers, pointing to a tissue-specific drug effect. Further studies are required to fully assess the relevance of VPA-induced Glut1 mRNA reduction to cerebral glucose homeostasis and the consequences of these changes for the drug's therapeutic and adverse effects. Moreover, although several drugs have been shown to affect the results of ^18^F-FDG-PET (Theodore et al., 1986a,b, 1989; Spanaki et al., 1999), little is known on the effects of medications on cerebral GLUT1 expression and on cerebral uptake carriers in general. Studying such drug effects can improve our understanding of the mechanisms of action of many compounds, including those that do not cross the BBB but can yet affect cerebral endothelial cells.

## Author contributions

AM designed and preformed all of the experiments, analyzed the data, and wrote the manuscript. SE, TB-H, and DE supervised the work and manuscript writing. AH contributed to study design and critically reviewed the manuscript.

### Conflict of interest statement

The authors declare that the research was conducted in the absence of any commercial or financial relationships that could be construed as a potential conflict of interest.

## Abbreviations

BBB: blood-brain barrier
G6P: glucose-6-phosphate
PET: positron emission tomography
VPA: valproic acid.
