# Supplementary material for: Effects of Valproic Acid on Cerebral Nutrient Carriers' Expression in the Rat
Source: Front Pharmacol. 2018 Sep 21;9:1054. doi: 10.3389/fphar.2018.01054 (PMC6160718; doi:10.3389/fphar.2018.01054)
Supplement: Supplementary file 1 [file Table_1.docx]

**Supporting Table 1. Genes, NCBI accessions and sequences recognizable by the probes used for the gene panel analysis (nCounter).**

| **Gene** | **Accession** | **Target Sequence** |
| --- | --- | --- |
| Abcb1a | NM_133401.1 | AAGCATCCGTGGGCCACATGATCAAGACGGGGAACTTAGCACCAAAGAGGCTCTGGATGACGACGTACCTCCAGCTTCCTTTTGGCGGATCCTGAAGTTG |
| Abcc1 | NM_022281.2 | TGTGTACTCACACTTCAATGAGACCTTGCTGGGGGTCAGTGTCATCCGTGCCTTTGAGGAACAGGAGCGCTTCATTCGCCAAAGTGACCTGAAAGTAGAT |
| Abcc2 | NM_012833.1 | ACAGTGACAAGATAATGGTCCTAGACAACGGGAAGATTGTCGAGTATGGCAGTCCTGAAGAACTGCTGTCCAACAGAGGTTCCTTCTATCTGATGGCCAA |
| Abcg2 | NM_181381.2 | AACAAGACAGAAGAGCCTTCCAAGAGAGAGAAGCCAATAATAGAAAATTTAGCCGAGTTTTATATCAACTCCACCATCTATGGAGAAACAAAAGCTGAAT |
| Actb | NM_031144.2 | CCCGCGAGTACAACCTTCTTGCAGCTCCTCCGTCGCCGGTCCACACCCGCCACCAGTTCGCCATGGATGACGATATCGCTGCGCTCGTCGTCGACAACGG |
| basigin | NM_001109882.1 | CTCTGTCCAGGAAGTTGACTCCAAGACACAGCTTACCTGCTTTTTGAACAGCAGTGGCATTGACATCGTTGGCCACCGCTGGATGAGAGGTGGCAAGGTA |
| Cdh1 | NM_031334.1 | CTATGCAAGCTGCGAAGATTCTAAGGTGCACACCTGATTCTTAGGCAGATGCCATAGTGAGATATGTTGCTTTGGTTCTCTATCCAATGCTGTGACCGGG |
| Cldn5 | NM_031701.2 | CAGGCTCTTGTGAGGACTTGACCGACCTTTTCTTCTATGCGCAGTTGGCCACGACATGGTGGAACTCTAAGATTTCATCGGTGAAGTAGCCACCAAACTG |
| Ednrb | NM_017333.1 | ATGGGAATGAATGAAGCCTCGGGAAAGCACTTAGATTCTTAGTCAGCACTTCAGCACGGCTCTTAAAAGCCCTCACTGCACTCACAGCCCACTTACATTT |
| Folr1 | NM_133527.2 | TCGGGGCATAATGAGTGCCCTGTGGGAGCCTCCTGCCATCCCTTCACTTTCTACTTCCCTACACCTGCTGTTCTGTGTGAGAAAATCTGGAGTCACTCCT |
| Gapdh | NM_017008.2 | TGGTGAAGGTCGGTGTGAACGGATTTGGCCGTATCGGACGCCTGGTTACCAGGGCTGCCTTCTCTTGTGACAAAGTGGACATTGTTGCCATCAACGACCC |
| Hprt1 | NM_012583.2 | AGCTTCCTCCTCAGACCGCTTTTCCCGCGAGCCGACCGGTTCTGTCATGTCGACCCTCAGTCCCAGCGTCGTGATTAGTGATGATGAACCAGGTTATGAC |
| Mmp9 | NM_031055.1 | TGCGTCGGGCGCTGCTCCAACTGCTGTATAAATATTAAGGTATTCAGTTACTCCTACTGGAAGGTATTATGTAACCATTTCTCTCTTACATCGGAGGACA |
| Ocln | NM_031329.2 | TCTTGGGAGCCTTGACATCTTGTTCATCATAAAGATCAGGTGACCAGTGACATCAGCCATGTCTGTGAGGCCTTTTGAGAGTCCACCTCCTTACAGGCCG |
| Slc16a1 | NM_012716.1 | TTTGGCTTTTGTTGATATGGTGGCCAGACCGTCCATGGGTCTTGCAGCCAACACCAGGTGGATCAGACCTCGAGTCCAGTACTTTTTTGCTGCTTCTGTT |
| Slc16a2 | NM_147216.1 | TCTTTTCCCTTCCTCATCAAAATGCTGGGAGACAGAATCAAGCTGGCCCAAACCTTCCAGGTGCTGAGTACCTTCATGTTTGTTCTTACGCTGCTCTCAC |
| Slc19a1 | NM_017299.2 | TTTCTGTATGTTCCAGATCCCGGACATCTGGGTCTGCTATGTGACCTTCGTGCTTTTCCGTGGGGCCTACCAGTTCCTTGTGCCCATTGCCACTTTTCAG |
| Slc1a1 | NM_013032.3 | CAGATACGAAGAAGTCTTACGTCAATGGGGGCTTCTCGGTAGACAAATCTGACACCATCTCGTTCACTCAGACCTCGCAGTTCTAGATGCCTGGCCTTAG |
| Slc22a1 | NM_012697.1 | AGAAAAGCGAAGTCCTTCGTTTGCCGACCTGTTCCGCACTCCCAACCTGAGGAAGCACACCGTCATCCTGATGTATCTATGGTTCTCTTGTGCTGTGCTG |
| Slc22a2 | NM_031584.1 | TTAATTGGCTACATCCTGATTACAGAATTTGTTGGGCTGGGCTATCGCAGAATGGTGGGGATTTGCTATCAAATCGCCTTCACCGTTGGCCTCCTGATCC |
| Slc22a3 | NM_019230.1 | CTAAGTGCAATGGGAAACACCTCTCGTCAAATTACTCAGAGATCACAGTTACAGATGAAGAGGTCAGTAACCCATCCTGTTTAGACCTTGTGAGGACTCC |
| Slc22a4 | NM_022270.1 | AAGATGAATGGCATCATGGCACCAGCAGTGATATTTGATCCTCTGGAGCTACAGGAGCTAAACTCCTTGAAGCAGCAGAAAGTCTTCATTCTGGACCTGT |
| Slc22a5 | NM_019269.1 | AACGGTCCTAAAGAGCACAGCCTTCTAACACCCTGTCCAGAAGGTGAAAAACTGAAAGGAAACCTGCGTGTAGTCAGAAATGCTCTCAATCACTGAGGGC |
| Slc22a6 | NM_017224.2 | GGCGCCACCTGCAGCTTGTGGTCTCTGTGCCTTTTTTCATTGCCTTCATCTACTCTTGGTTCTTCATTGAGTCAGCCCGCTGGTACTCCTCCTCAGGAAG |
| Slc22a8 | NM_031332.1 | TTGGAGTCAACATCTACATACTCCAGATTATCTTTGGTGGGGTTGACATCCCAGCCAAGTTCATCACAATCCTCTCCTTAAGTTATCTGGGCCGGCGCAT |
| Slc27a1 | NM_053580.2 | CGACAGCCGGAGCGCCTGGCGCTGGTAGATGCGAGTAGCGGTATCTGCTGGACCTTCGCACAGCTAGACACCTACTCCAATGCTGTGGCCAATCTGTTCC |
| Slc28a2 | NM_031664.1 | GGGAAACCTCCACTTCCTGCTTGTGAGAGTATTAATCCCTGCTGCAGCTGTCCATCCCCACTGACATCAGACTCCAGTTTCTCCAGGATCCAGTGTGTTC |
| Slc2a1 | NM_138827.1 | ATCGCTTTGGCAGGCGGAACTCCATGCTGATGATGAACCTGTTGGCCTTTGTGTCTGCCGTGCTTATGGGTTTCTCCAAACTGGGCAAGTCCTTTGAGAT |
| Slc3a2 | NM_019283.1 | GAAGTCAATGAAACCGACTTGAAACAGATTGATCCCGATTTAGGCTCCCAGGAAGATTTTAAAGACCTTCTACAAAGTGCCAAGAAAAAGAGCATTCACA |
| Slc44a1 | NM_053492.3 | CTGGATAATGACACTTCTCTTCCTTGGCACTACTGGCAGTGCTGTTCAGAATGAACAAGGTTTCGTGGAGTACAAAATCTCTGGGCCTTTGCAGTACATG |
| Slc44a2 | NM_001134715.1 | CTACCCACTGGTGACTTTCTTCCTCTTGTGTCTCTGCATTGCCTACTGGGCCAGCACTTCTGTCTTCCTGTCTACCTCTAATGTAGCCGTGTACAAGATT |
| Slc46a1 | NM_001013969.1 | GTTATGACTCTATATGCAGCATTCTGTTTTGGTGAGACAGTGAAGGAGCCAAAGTCCACGAGGCTTTTCACGCTCCGCCATCACCGATCCATTGTCCAGC |
| Slc6a14 | NM_001037544.1 | AACTTCCCAGTGAACAGTATTGGGATAAAGTGACGCTTCGGAGGTCAAGTGGAATGGATGAAACTGGCGTAATTGTGTGGTACTTAGCACTTTGCCTTCT |
| Slc7a5 | NM_017353.1 | CCACCTGGGAGTCATGTCCTGGATCATTCCTGTCTTCGTGGGCTTGTCCTGCTTCGGCTCTGTCAATGGGTCTCTGTTCACGTCCTCAAGACTGTTCTTC |
| Slc7a8 | NM_053442.1 | ATCAAGATCAGCCTGCTGTTTCCCATCATCTACTTGCTGTTCTGGGCCTTCCTGCTGATTTTCAGCCTGTGGTCAGAGCCAGTAGTATGCGGCATTGGCC |
| Slco1b2 | NM_031650.1 | TATGCAAAAGAAAACGACATTGGCTCTCTAGGCAACTCTACATTGACCTGTTTCATCAATCAAATGACATCACCCACTGGACCTTCACCTGAGATAGTGG |
| Slco1c1 | NM_053441.1 | GTGAAGCTACAAAACTCTACTTGGGATCATCTGTCTTTGGCTACCTCCTCTTCCTCTCTCTGTTTGCACTGGGATGTGAAAATTCCAGTGTGGCCGGACT |
| Slco2b1 | NM_080786.1 | GCCACTCTAGCTTTTGAGAATAGAATCCTAGTCCATATTCCCGGGGCTTTGTAGCTAAGACCTGATACTGTCATAGCCAAGTCATGGGTGACCTCTGTTT |
| Tjp1 | NM_001106266.1 | GACAGCTACAGGAAAATGACCGAGTCGCAATGGTTAACGGAGTTTCAATGGATAATGTTGAACATGCTTTTGCTGTTCAGCAGCTAAGGAAAAGTGGGAA |
| Slco1a5 | NM_030838.1 | CTCTGGGACCAACATAGTAGGCCAGAACCAACTTTTTCCAGTTGATCTTCTCCCTTTACTCCTGTACTGTGGTGAATATGTCCTACCACAAACAAACAAA |
