# Supplementary material for: Effects of Valproic Acid on Cerebral Nutrient Carriers' Expression in the Rat
Source: Front Pharmacol. 2018 Sep 21;9:1054. doi: 10.3389/fphar.2018.01054 (PMC6160718; doi:10.3389/fphar.2018.01054)
Supplement: Supplementary file 2 [file Data_Sheet_1.docx]

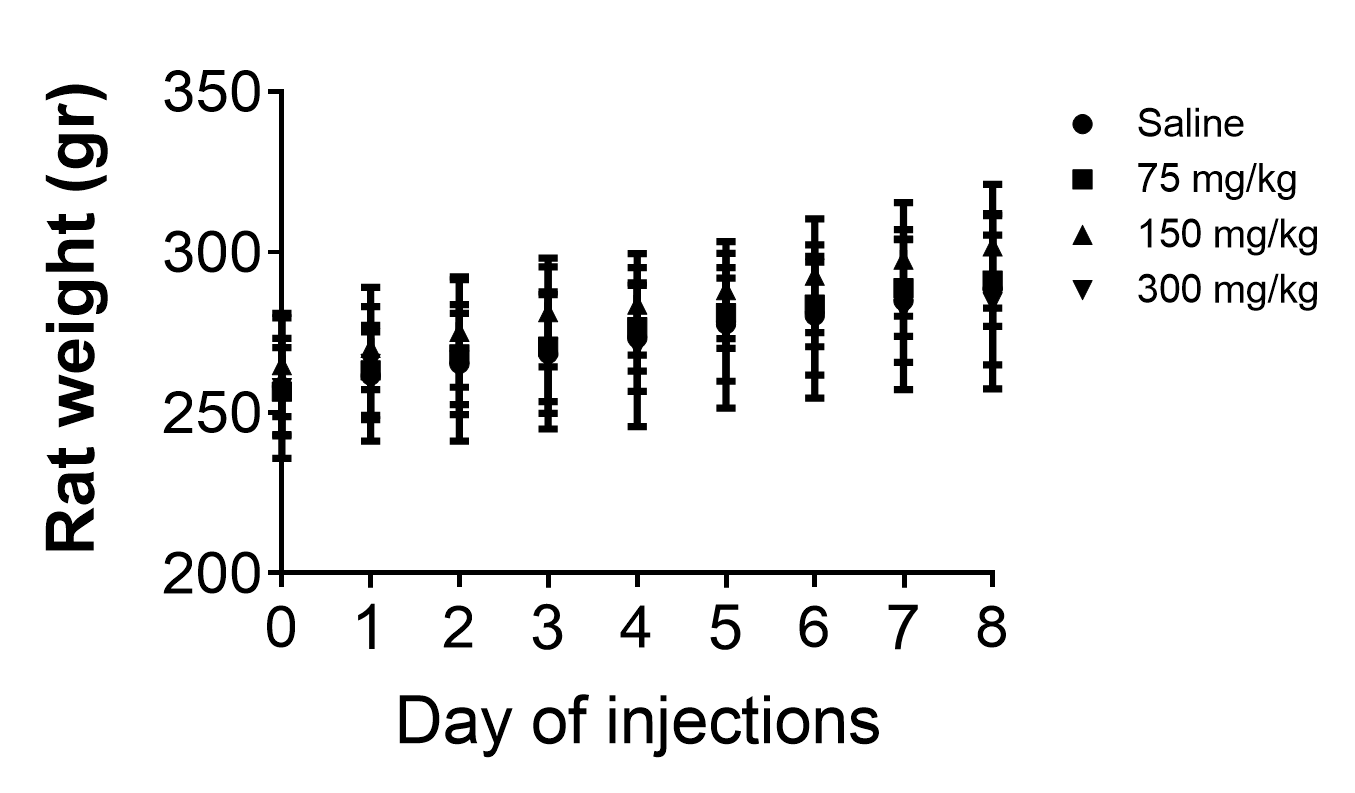


**Supporting Figure 1.** Effect of VPA on Rats' weight. Average weight (gr) of rats from each experimental group, on each of the days of injection. N = 6-7.
